# Detection of somatic changes in human cancer DNA by DNA fingerprint analysis.

**DOI:** 10.1038/bjc.1987.71

**Published:** 1987-04

**Authors:** S. L. Thein, A. J. Jeffreys, H. C. Gooi, F. Cotter, J. Flint, N. T. O'Connor, D. J. Weatherall, J. S. Wainscoat

## Abstract

**Images:**


Br. J. Cancer (1987), 55, 353–356 © The Macmillan Press Ltd., 1987

### Detection of somatic changes in human cancer DNA by DNA fingerprint analysis

S.L. Thein[1], A.J. Jeffreys[2], H.C. Gooi[3], F. Cotter[4], J. Flint[1], N.T.J. O'Connor[1], D.J. Weatherall[1] & J.S. Wainscoat[5]

[1]*MRC Molecular Haematology Unit, John Radcliffe Hospital, Oxford;* [2]*Department of Genetics, University of Leicester;* [3]*Yorkshire Regional Blood Transfusion Centre, Leeds;* [4]*Department of Medical Oncology, St. Bartholomew's, London and* [5]*Department of Haematology, John Radcliffe Hospital, Oxford, UK.*

**Summary** Minisatellite DNA probes which can detect a large number of autosomal loci dispersed throughout the human genome were used to examine the constitutional and tumour DNA of 35 patients with a variety of cancers of which eight were of gastrointestinal origin. Somatic changes were seen in the tumour DNA in ten of the 35 cases. The changes included alterations in the relative intensities of hybridising DNA fragments, and, in three cases of cancers of gastrointestinal origin, the appearance of novel minisatellite fragments not seen in the corresponding constitutional DNA. The results of this preliminary study suggests that DNA fingerprint analysis provides a useful technique for identifying somatic changes in cancers.

It is now widely accepted that tumours arise through the accumulation of several genetic changes affecting the control of cell proliferation (Klein & Klein, 1985). Over 30 different cellular loci presumably involved in these pathways have now been identified (Barbacid, 1986). These genes have been designated oncogenes whereas their counterparts in normal cells are referred to as proto-oncogenes. Activation of the cellular oncogenes is thought to be an important step in the development of neoplasia, and the somatic change which causes this activation can be a gross chromosomal translocation or just a single point mutation (Barbacid, 1986). Somatic changes which result from major chromosomal rearrangements are detectable by karyotype analysis (Sandberg, 1980; Yunis, 1983) although chromosomal preparations from solid tumours can be technically difficult. DNA analysis can also detect somatic variations; in particular, chromosomal loss or the development of somatic homozygosity can now be demonstrated by restriction fragment length polymorphism (RFLP) analysis using single copy gene probes (Wainscoat & Thein, 1985). This approach is ideal for the study of tumours such as retinoblastoma (Cavenee *et al.*, 1983) or Wilms' tumour (Koufos *et al.*, 1984; Orkin *et al.*, 1984; Reeve *et al.*, 1984) in which there is a consistent loss of a particular chromosomal region. For other tumours a battery of DNA probes representing different loci may be used to detect chromosomal loss (Dracopoli *et al.*, 1985).

We describe here an alternative approach for screening the human genome for somatic changes in cancers using DNA fingerprint analysis. DNA fingerprints represent multiple hypervariable fragments derived from a large number of autosomal loci dispersed throughout the human genome and show both somatic and germline stability (Jeffreys *et al.*, 1985*a*, *b*, 1986). We have analysed the constitutional and tumour DNA of 35 cancer patients and observed somatic changes in the tumour DNA in ten cases. The changes include alterations in the relative intensities of hybridising DNA fragments, and, in three cases of cancers of gastrointestinal origin, the generation of novel minisatellite fragments not seen in the corresponding constitutional DNA.

### Materials and methods

### *Patients*

Thirty-five patients with a variety of cancers were studied (Table I). The diagnoses of the solid tumours were made by histological examination and the cases of myelodysplasia (MDS), all of which had karyotypic abnormalities, fulfilled the FAB classification (Bennett *et al.*, 1982). All tissues and peripheral blood leucocytes were obtained before chemotherapy or radiotherapy. DNA was isolated from tumour tissues (representing tumour DNA), from peripheral blood leucocytes (representing constitutional DNA) and, in some cases, from adjacent normal tissue. In the MDS patients, tumour DNA was obtained from bone marrow or peripheral blood leucocytes and constitutional DNA from Epstein–Barr virus (EBV)-transformed B-cells.

### *DNA Analysis*

DNA was isolated from peripheral blood leucocytes by proteinase-K and phenol-chloroform extraction as described (Old & Higgs, 1983). The fresh solid tissues were dissected free of fat, minced and digested overnight at 37°C in 0.5% SDS and proteinase-K; DNA was subsequently isolated by phenol-chloroform extraction as for the leucocytes. Equivalent amounts of constitutional or tumour DNA (8–10 $\mu$g) from each individual were digested with *Hinf*I, *Alu*I and *Hae*III under conditions recommended by the manufacturers (Boehringer Mannheim), in the presence of spermidine trichloride and recovered by ethanol precipitation. The resultant DNA fragments of the constitutional and tumour DNA of each patient were electrophoresed in adjacent tracks through a 22 cm long 1.0% agarose gel at 35 V for $\sim$36 h at room temperature until all DNA fragments $<$2 kb long had electrophoresed off the gel. The separated DNA fragments were transferred to a Schleicher and Schull nitrocellulose membrane (BA 85) and hybridised to $^{32}$P-labelled minisatellite probe 33.15 or 33.6 as described elsewhere (Jeffreys *et al.*, 1985*a*, *b*). After hybridisation, filters were washed for 1 h in 1 $\times$ SSC at 65°C and autoradiographed without intensifying screens for 3–4 days at $-70$°C.

### Results

The tumour and constitutional DNA fingerprints were indistinguishable in the majority (25/35) of cancer patients studied (Figure 1, Table I). In nine cases, including some myelomas, breast carcinomas and gastro-intestinal tract carcinomas, there were shifts in the relative intensities of hypervariable DNA fragments in the tumour DNA when compared to the constitutional DNA. In two cases (patients AC and FB), the shifts in band intensities were also seen in DNA fingerprints produced after digestion with *Alu*I or *Hae*III confirming that these changes were not due to tumour-specific DNA methylation changes which could

Correspondence: S.L. Thein.
Received 21 October 1986; and in revised form, 4 December 1986.

**Table I** Comparison of tumour and constitutional DNA fingerprints in 35 human cancers

| Tumour | Patient | | DNA source | Changes in tumour DNA fingerprint: Intensity shift | Changes in tumour DNA fingerprint: New bands |
|---|---|---|---|---|---|
| Lymphoma | | | | | |
| T-cell | 1 | PH | LN, PB | − | − |
| | 2 | LS | LN, PB | − | − |
| | 3 | $W_1$ | LN, PB | − | − |
| B-cell | 4 | $C_1$ | LN, PB | − | − |
| | 5 | $C_2$ | LN, PB | − | − |
| | 6 | $T_1$ | pleural fluid, PB | − | − |
| Multiple myeloma | 7 | EL | BM, PB | + | − |
| | 8 | $T_2$ | BM, PB | − | − |
| | 9 | $C_3$ | BM, PB | + | − |
| | 10 | $H_1$ | BM, PB | − | − |
| | 11 | $W_2$ | BM, PB | − | − |
| | 12 | G | BM, PB | − | − |
| | 13 | O | BM, PB | + | − |
| Waldenstrom's disease | 14 | $D_1$ | BM, PB | − | − |
| Hodgkin's disease | 15 | $H_2$ | LN., PB | − | − |
| Myelodysplasia | 16 | K | BM, PB, CL | − | − |
| | 17 | $C_4$ | PB, CL | − | − |
| | 18 | $H_3$ | PB, CL | − | − |
| | 19 | $D_2$ | PB, CL | − | − |
| | 20 | B | PB, CL | − | − |
| | 21 | $R_2$ | PB, CL | − | − |
| | 22 | U | PB, CL | − | − |
| | 23 | L | PB, CL | − | − |
| Breast carcinoma | 24 | J | T, PB | − | − |
| | 25 | $R_1$ | T, PB | + | − |
| | 26 | EA | T, N, PB | + | − |
| | 27 | AG | T, N, PB | − | − |
| Gastric carcinoma | 28 | HT | T, N, PB | − | − |
| | 29 | B | T, N, PB | + | − |
| Gastro-oesphageal carcinoma | 30 | AC | T, N, PB | + | + |
| Colonic carcinoma | 31 | BA | T, N, PB | − | − |
| | 32 | WM | T, N, PB | + | − |
| | 33 | GS | T, PB | − | − |
| Rectal carcinoma | 34 | PW | T, N, PB | − | + |
| | 35 | FB | T, PB | + | + |

Constitutional DNA was represented by DNA from peripheral blood leucocytes (PB) and in some breast and gastro-intestinal carcinomas from adjacent normal (N) tissue. Tumour DNA was represented by DNA from lymph-node (LN), cancerous cells in pleural fluid in case No. 6 (patient $T_1$), bone marrow (BM) and tumour (T) tissue. In myelodysplasia, tumour DNA was represented by BM or PB and constitutional DNA by EBV-transformed B lymphocytes (CL). The symbol (+) indicates presence and (−) absence of changes in tumour DNA fingerprint. The changes in the tumour DNA fingerprints in patients AC, PW and FB seen after *Hinf*I digestion, were also produced after digestion with *Alu*I or *Hae*III. Shifts in band intensities alone in seven cases (patients EL, C3, O, $R_1$, EA, B and WM) might, in part at least, be related to tumour-specific DNA methylation changes (Goelz *et al.*, 1985) which could affect *Hinf*I cleavage sites.

affect *Hinf*I cleavage sites and therefore hypervariable DNA fragment length (*Hinf*I sites terminating in mCG, GANTmCG, are resistant to *Hinf*I cleavage; McClelland & Nelson, 1985). In addition, in patient AC where adjacent normal tissue was available for study, the changes were present only in tumour tissue but not in the adjacent normal tissue showing that these changes were specific to tumour rather than tissue.

Three patients (AC, PW and FB, Table I, Figure 2a) showed new bands in the tumour DNA fingerprint. Patients PW and FB had moderately differentiated adenocarcinoma of the rectum and AC had an undifferentiated adenocarcinoma of the gastro-oesophageal junction. Patient PW showed a novel 14.4 kb band in the tumour DNA fingerprint which was not present in the constitutional DNA fingerprints of both peripheral blood and adjacent normal tissue. In addition, the hybridisation signal of a larger 16.2 kb fragment was approximately halved in tumour DNA as compared to constitutional DNA. No other differences were noted in the tumour DNA fingerprint. Patient FB showed two types of changes in the tumour DNA, the presence of a new band of approximately 10.5 kb and a reduction in the hybridisation intensity in four bands of approximate sizes 5.7, 5.0, 4.0 and 3.1 kb. Patient AC had a novel 4.6 kb fragment, reduced hybridisation signal of a 4.4 kb band and an increased signal of a 2.8 kb band in the tumour DNA fingerprint. These changes in the tumour DNA were confirmed by hybridisation of probe 33.15 to DNA digested with *Alu*I and *Hae*III (Figure 2b). Furthermore, the changes occurred in bands of similar sizes, as expected since *Hinf*I, *Alu*I and *Hae*III, which cleave at 4 bp sequences that are unlikely to be present in the tandem repeat sequence, should each release a minisatellite in a similar sized fragment. Repeat hybridisation of *Hinf*I, *Alu*I and *Hae*III digested

**Figure 1** Representative constitutional and tumour DNA fingerprints of human cancers. The tumour types are shown above the autoradiographs. Lanes C and $C_1$ represent constitutional DNA from peripheral blood leucocytes or EBV-transformed lymphocytes; $C_2$, constitutional DNA from adjacent normal tissue, and lanes T, tumour DNA. MM – multiple myeloma, MDS – myelodysplasia, Ca colon – carcinoma of the colon and breast – breast carcinoma. Note that the tumour and constitutional DNA fingerprints in these cases are indistinguishable.

DNAs of patients PW, FB and AC with minisatellite probe 33.6, which detects hypervariable DNA fragments derived from a different set of loci (Jeffreys *et al.*, 1986), did not show any differences between tumour and constitutional DNA fingerprints (data not shown).

### Discussion

Cancer is a multistep process that probably results from an accumulation of a series of somatic changes. DNA fingerprint analysis provides a useful new approach for studying some of these somatic changes in tumour DNA. A number of processes could result in the alteration of DNA fingerprints. For example, loss of chromosomes or chromosomal regions through deletion, mitotic nondisjunction or mitotic recombination would lead to loss of associated minisatellite fragments. Conversely, localised amplification of DNA (Stark & Wahl, 1984; Schimke, 1984) including a minisatellite would cause specific band intensification. Tissue- or tumour-specific changes in DNA methylation (Goelz *et al.*, 1985) could also affect DNA fingerprints; the latter can be excluded by using restriction enzymes such as *Alu*I and *Hae*III the cleavage sites of which are not blocked by CpG methylation. Of considerable interest is the appearance of novel fragments in tumour DNA fingerprints. These bands presumably arise by length changes of pre-existing minisatellites, perhaps by unequal sister-chromatid exchange. Thus the novel 14.4 kb band in patient PW appears to have arisen by contraction of the larger 16.2 kb fragment which is still present although at reduced intensity in the tumour DNA fingerprint. The 1.8 kb deletion relationship between the larger and mutant bands is also seen in *Alu*I and *Hae*III digests, as expected if the 16.2 kb band is the precursor of the 14.4 kb mutant fragment. The retention of the parental fragment in tumour DNA suggests that the tumour is comprised of a mixed population of parental and mutant cells; the alternative explanation that PW is homozygous for

**Figure 2** (a) Comparison of the constitutional and tumour DNA fingerprints produced after *Hinf*I digestion in 3 cancer patients. The patients' initials are shown above the blots and correspond to details given in **Table I.** Lanes 1 represent constitutional DNA from peripheral blood leucocytes, lanes 2, tumour DNA and lanes 3, constitutional DNA from adjacent normal tissue. The symbol ◀ indicates the position of novel bands and shifts in band intensities seen in the tumour DNA fingerprints, AC – 4.6, 4.4, 2.8 kb, PW – 16.2, 14.4 kb and FB – 10.5, 5.7, 5.0, 4.0, 3.1 kb. These changes were also seen after digestion with *Alu*I or *Hae*III. (b) Comparison of the tumour and constitutional DNA fingerprints in patient PW. Lanes 1 – constitutional DNA from peripheral blood leucocytes, lanes 2 – tumour DNA, lanes 3 – constitutional DNA from adjacent normal tissue. The symbol ◀ indicates the position of the novel band present in tumour DNA after digestion with *Hinf*I, *Alu*I, and *Hae*III. Its probable progenitor band is marked ◁ .

the 16.2 kb fragment and that the tumour is heterozygous for the new mutant band is less likely in view of the high level of heterozygosity of large minisatellite fragments (Jeffreys *et al.*, 1985*b*).

Thirty-five different tumours of which eight were of gastro-intestinal origin have been studied. Mutant bands in tumour DNA fingerprints were observed in three cases, all of gastro-intestinal origin (two rectal and one gastro-oesophageal carcinoma). In two cases where adjacent normal tissue was available for study, no changes were seen on comparing normal tissue and blood DNA fingerprints. This suggests that the somatic mutations seen are tumour specific rather than tissue specific. However, it is not clear whether these novel bands are related to the pathogenesis of the tumour, or whether they are the products of unequal mitotic recombination events which arise in all tissues but only become apparent on clonal expansion of a malignant cell. In any event, such changes even though not directly related to the tumour phenotype could provide novel markers for studying tumour clonality and tumour progression. Also, it is presently not possible to determine whether the somatic mutations observed in the three gastro-intestinal tumours involve the same locus. It should be possible to clone the clearly-resolved mutant minisatellite from patient PW to provide a locus-specific probe (Wong *et al.*, 1986) suitable for studying the tumours from patients FB and AC which should help to resolve the issue of whether the genomic rearrangements in the tumours of patients PW, FB and AC involve a common chromosomal region.

Recently, an oncogene (*onc*-D or *trk*) has been isolated from the DNA of a human carcinoma of the colon and was shown to have arisen by recombination between two separate loci (Martin-Zanca *et al.*, 1986). It is possible that an enhanced level of recombination occurs in many tumours, rearranging both oncogenes and minisatellites. Some of these rearrangements are detectable by karyotype analysis e.g. generation of the Philadelphia chromosome in chronic myeloid leukaemia (Heisterkamp *et al.*, 1983) and translocation of the c-*myc* oncogene in Burkitt's lymphoma (Yunis, 1983). In view of the evidence that the majority of the cancers show substantial genetic rearrangement it might have been expected that a higher proportion of the tumours analysed would have shown differences in the DNA fingerprints as compared to the constitutional DNA. One explanation for this unexpected finding is that the DNA fingerprints obtained from the minisatellite probe 33.15 are derived from an estimated number of 30 loci (Jeffreys *et al.*, 1986) and therefore will not detect aneuploidy or hemizygosity over a large proportion of the human genome. Nevertheless, DNA fingerprinting analysis offers another approach to the detection of previously uncharacterised genomic rearrangements. Locus-specific hybridisation probes could be prepared from a mutant minisatellite and then used both to determine the frequency of genomic rearrangements at this locus in other cancers and to localise the minisatellite within the human genome. This approach is currently being tested by cloning the mutant minisatellite from patient PW.

We thank Linda Roberts and Liz Gunson for preparation of the manuscript. We are grateful to Victoria Wilson and Zilla Wong for technical assistance. SLT is a Wellcome Senior Research Fellow in Clinical Science. AJJ is a Lister Institute Research Fellow. This work was supported in part by a grant to AJJ from the Medical Research Council. JSW is supported in part by the Cancer Research Campaign. The minisatellite probes are the subject of patent applications. Commercial enquiries should be addressed to the Lister Institute of Preventative Medicine, Brockley Hill, Stanmore, Middlesex, U.K.

### References

BARBACID, M. (1986). Mutagens, oncogenes and cancer. *Trends Genet.*, **2**, 188.

BENNETT, J.M., CATOVSKY, D., DANIEL, M.T. & 2 others (1982). Proposals for the classification of the myelodysplastic syndromes. *Br. J. Haematol.*, **51**, 189.

CAVENEE, W.K., DRYJA, T.P., PHILLIPS, R.A. & 6 others (1983). Expression of recessive alleles by chromosomal mechanisms in retinoblastoma. *Nature*, **305**, 779.

DRACOPOLI, N.C., HOUGHTON, A.N. & OLD, L.J. (1985). Loss of polymorphic restriction fragments in malignant melanoma: implications for tumour heterogeneity. *Proc. Natl Acad. Sci., USA*, **82**, 1470.

GOELZ, S.E., VOGELSTEIN, B., HAMILTON, S.R. & FEINBERG, A.P. (1985). Hypomethylation of DNA from benign and malignant colon neoplasms. *Science*, **228**, 187.

HEISTERKAMP, N., STEPHENSON, J.R., GROFFEN, J. & 4 others (1983). Localization of the c-abl oncogene adjacent to a translocation breakpoint in chronic myelocytic leukaemia. *Nature*, **306**, 239.

JEFFREYS, A.J., WILSON, V. & THEIN, S.L. (1985*a*). Hypervariable 'minisatellite' regions in human DNA. *Nature*, **314**, 67.

JEFFREYS, A.J., WILSON, V. & THEIN, S.L. (1985*b*). Individual-specific 'fingerprints' of human DNA. *Nature*, **316**, 76.

JEFFREYS, A.J., WILSON, V., THEIN, S.L., WEATHERALL, D.J. & PONDER, B.A.J. (1986). DNA 'fingerprints' and linkage analysis in human pedigrees. *Am. J. Hum. Genet.*, **39**, 11.

KLEIN, G. & KLEIN, E. (1985). Evolution of tumours and the impact of molecular oncology. *Nature*, **315**, 190.

KOUFOS, A., HANSON, M.F., LAMPKIN, B.C. & 4 others (1984). Loss of alleles at loci on human chromosome 11 during genesis of Wilms' tumour. *Nature*, **309**, 170.

MARTIN-ZANCA, D., HUGHES, S.H. & BARBACID, M. (1986). A human oncogene formed by the fusion of truncated tropomyosin and protein tyrosine kinase sequences. *Nature*, **319**, 743.

McCLELLAND, M. & NELSON, M. (1985). The effect of site specific methylation on restriction endonuclease digestion. *Nucleic Acids Res.* (suppl), **13**, r201.

OLD, J.M. & HIGGS, D.R. (1983). Gene analysis. In *Methods in Haematology*, Weatherall, D.J. (ed) Vol. **6**, p. 74. Churchill Livingstone: Edinburgh.

ORKIN, S.H., GOLDMAN, D.S. & SALLAN, S.E. (1984). Development of homozygosity for chromosome 11p markers in Wilms' tumour. *Nature*, **309**, 172.

REEVE, A.E., HOUSIAUX, P.J., GARDNER, R.J.M., CHEWINGS, W.E., GRINDLEY, R.M. & MILLOW, L.J. (1984). Loss of a Harvey ras allele in Sporadic Wilms' tumour. *Nature*, **309**, 174.

SNADBERG, A.A. (1980). *The Chromosome in Human Cancer and Leukemia.* Elsevier: New York.

SCHIMKE, R.T. (1984). Gene amplification in cultured animal cells. *Cell*, **37**, 705.

STARK, G.R. & WAHL, G.M. (1984). Gene amplification. *Ann. Rev. Biochem.*, **53**, 447.

WAINSCOAT, J.S. & THEIN, S.L. (1985). Polymorphism in human DNA: application to cancer studies. *Trends Biochem. Sci.*, **10**, 474.

WONG, W., WILSON, V., JEFFREYS, A.J. & THEIN, S.L. (1986). Cloning a selected fragment from a human DNA 'fingerprint': isolation of an extremely polymorphic minisatellite. *Nucleic Acids Res.*, **14**, 4605.

YUNIS, J.J. (1983). The chromosomal basis of neoplasia. *Science*, **221**, 227.


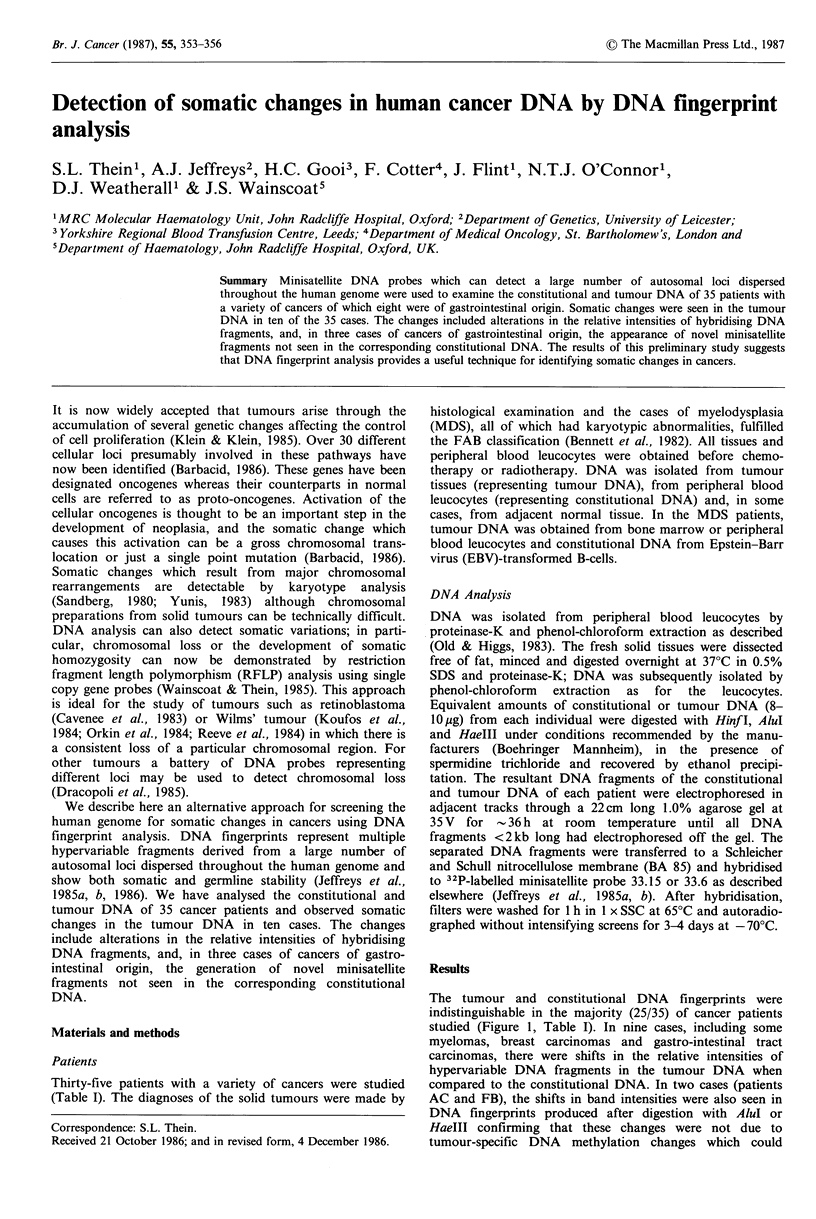

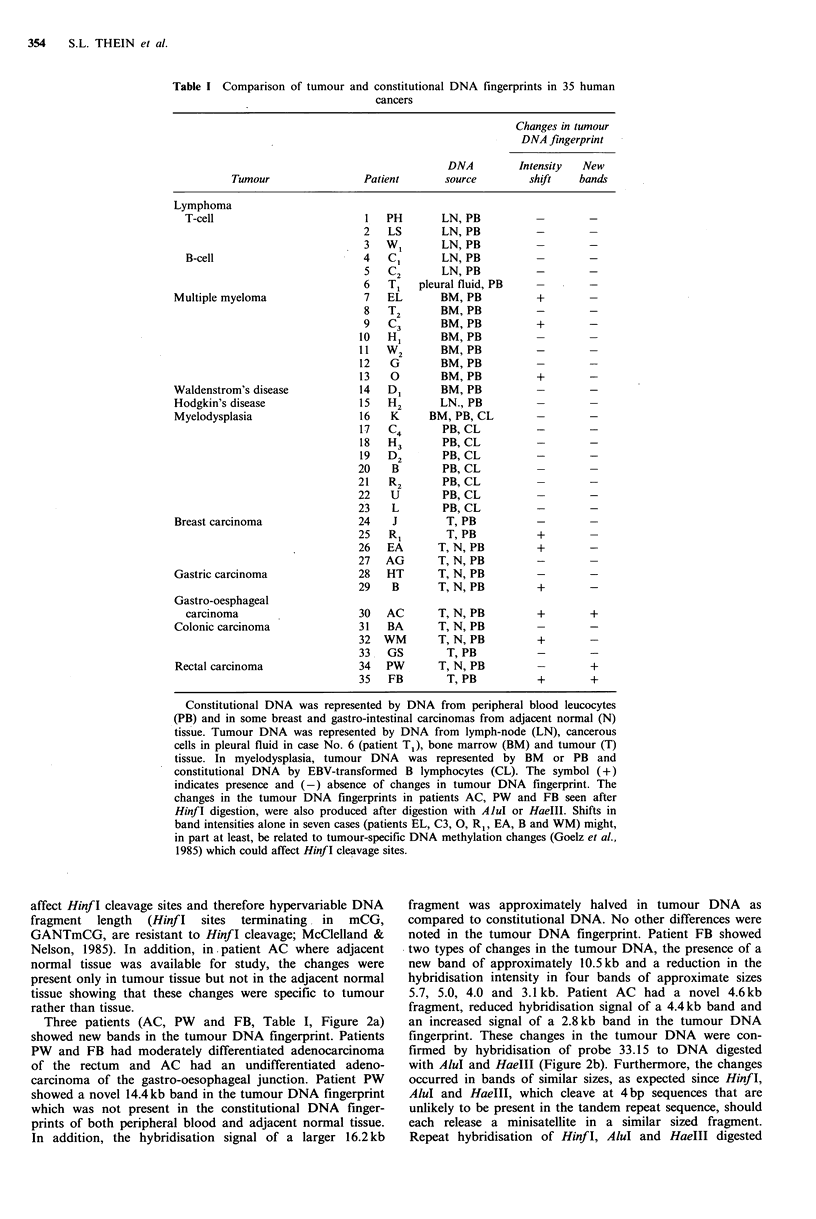

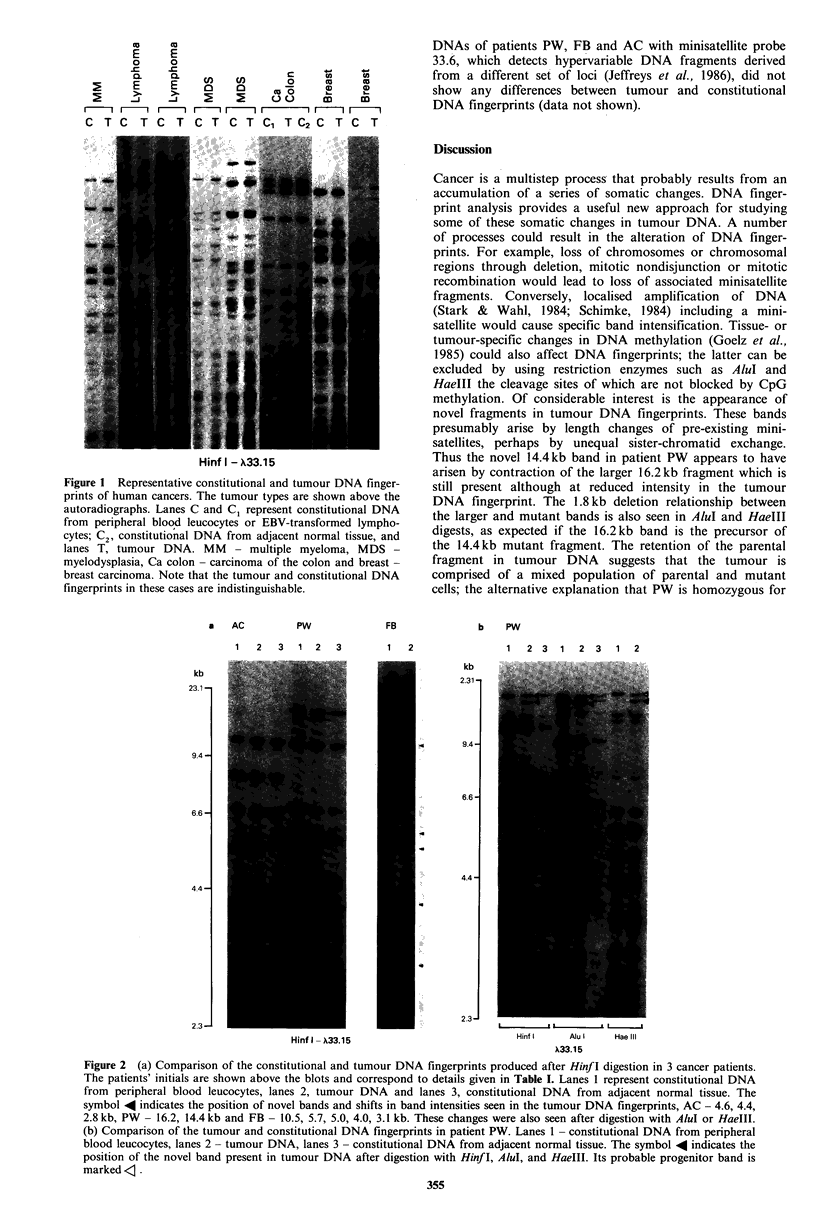

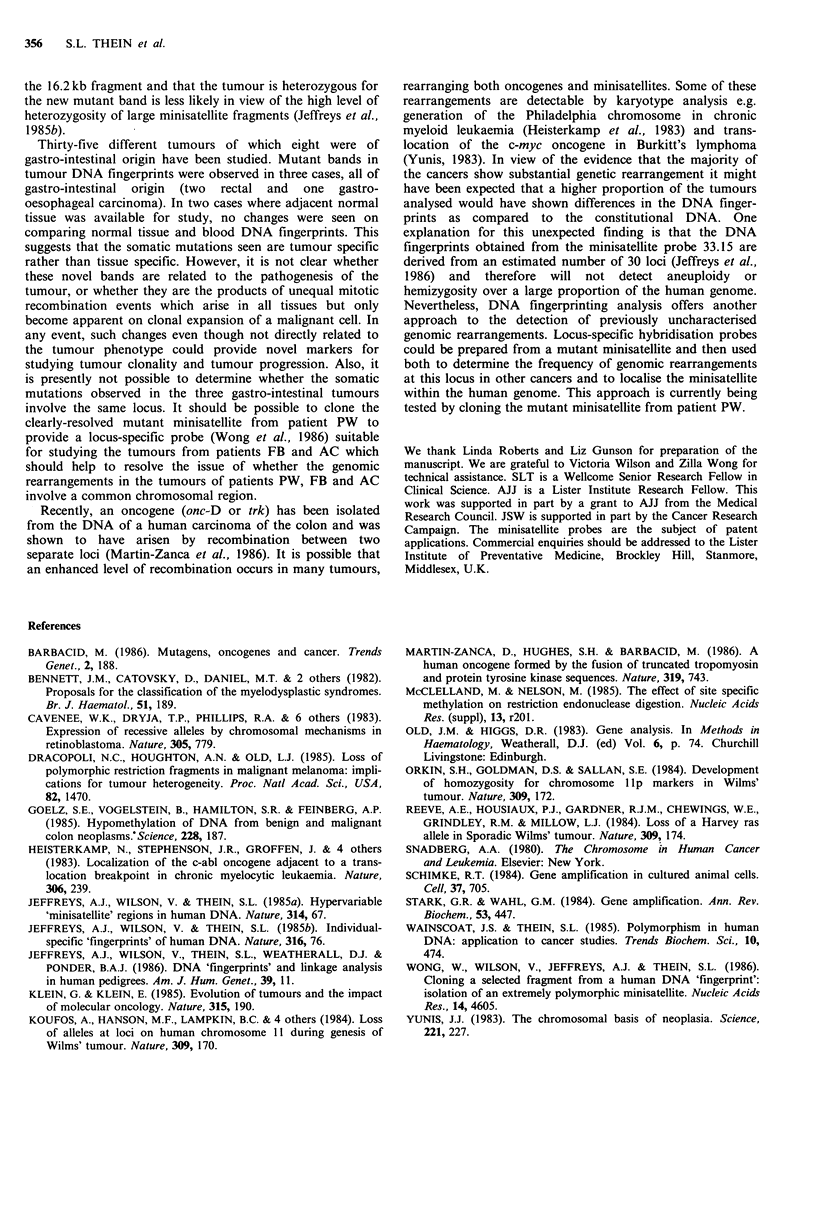


## References

[OCR_00443] Bennett J. M., Catovsky D., Daniel M. T., Flandrin G., Galton D. A., Gralnick H. R., Sultan C. (1982). Proposals for the classification of the myelodysplastic syndromes.. Br J Haematol.

[OCR_00448] Cavenee W. K., Dryja T. P., Phillips R. A., Benedict W. F., Godbout R., Gallie B. L., Murphree A. L., Strong L. C., White R. L. Expression of recessive alleles by chromosomal mechanisms in retinoblastoma.. Nature.

[OCR_00453] Dracopoli N. C., Houghton A. N., Old L. J. (1985). Loss of polymorphic restriction fragments in malignant melanoma: implications for tumor heterogeneity.. Proc Natl Acad Sci U S A.

[OCR_00459] Goelz S. E., Vogelstein B., Hamilton S. R., Feinberg A. P. (1985). Hypomethylation of DNA from benign and malignant human colon neoplasms.. Science.

[OCR_00464] Heisterkamp N., Stephenson J. R., Groffen J., Hansen P. F., de Klein A., Bartram C. R., Grosveld G. (1983). Localization of the c-ab1 oncogene adjacent to a translocation break point in chronic myelocytic leukaemia.. Nature.

[OCR_00470] Jeffreys A. J., Wilson V., Thein S. L. (1985). Hypervariable 'minisatellite' regions in human DNA.. Nature.

[OCR_00474] Jeffreys A. J., Wilson V., Thein S. L. (1985). Individual-specific 'fingerprints' of human DNA.. Nature.

[OCR_00478] Jeffreys A. J., Wilson V., Thein S. L., Weatherall D. J., Ponder B. A. (1986). DNA "fingerprints" and segregation analysis of multiple markers in human pedigrees.. Am J Hum Genet.

[OCR_00483] Klein G., Klein E. (1985). Evolution of tumours and the impact of molecular oncology.. Nature.

[OCR_00487] Koufos A., Hansen M. F., Lampkin B. C., Workman M. L., Copeland N. G., Jenkins N. A., Cavenee W. K. (1984). Loss of alleles at loci on human chromosome 11 during genesis of Wilms' tumour.. Nature.

[OCR_00492] Martin-Zanca D., Hughes S. H., Barbacid M. A human oncogene formed by the fusion of truncated tropomyosin and protein tyrosine kinase sequences.. Nature.

[OCR_00497] McClelland M., Nelson M. (1985). The effect of site specific methylation on restriction endonuclease digestion.. Nucleic Acids Res.

[OCR_00507] Orkin S. H., Goldman D. S., Sallan S. E. (1984). Development of homozygosity for chromosome 11p markers in Wilms' tumour.. Nature.

[OCR_00512] Reeve A. E., Housiaux P. J., Gardner R. J., Chewings W. E., Grindley R. M., Millow L. J. (1984). Loss of a Harvey ras allele in sporadic Wilms' tumour.. Nature.

[OCR_00521] Schimke R. T. (1984). Gene amplification in cultured animal cells.. Cell.

[OCR_00525] Stark G. R., Wahl G. M. (1984). Gene amplification.. Annu Rev Biochem.

[OCR_00534] Wong Z., Wilson V., Jeffreys A. J., Thein S. L. (1986). Cloning a selected fragment from a human DNA 'fingerprint': isolation of an extremely polymorphic minisatellite.. Nucleic Acids Res.

[OCR_00540] Yunis J. J. (1983). The chromosomal basis of human neoplasia.. Science.

